# Process and outcome of outpatient psychotherapies under clinically representative conditions in Austria: protocol and feasibility of an ongoing study

**DOI:** 10.3389/fpsyt.2024.1264039

**Published:** 2024-03-06

**Authors:** Yvonne Schaffler, Andrea Jesser, Elke Humer, Katja Haider, Christoph Pieh, Thomas Probst, Brigitte Schigl

**Affiliations:** ^1^ Department for Psychosomatic Medicine and Psychotherapy, University for Continuing Education Krems, Krems, Austria; ^2^ Faculty of Psychotherapy Science, Sigmund Freud University Vienna, Vienna, Austria; ^3^ Division of Psychotherapy, Department of Psychology, Paris Lodron University Salzburg, Salzburg, Austria; ^4^ Department Psychology and Psychodynamics, Karl Landsteiner University of Health Sciences, Krems, Austria

**Keywords:** psychotherapy, longitudinal study, gender, therapeutic relationship, helping alliance, naturalistic outcome study, outpatient practice, psychotherapy effectiveness

## Abstract

**Background:**

While most studies assessing psychotherapy efficacy are randomized-controlled trials conducted in research institutions or short clinical treatments, the understanding of psychotherapy effectiveness under regular, clinically representative conditions, particularly in outpatient practice, remains limited. Representative data examining the effectiveness of psychotherapy under real-world conditions in Austria is lacking.

**Aims and Methods:**

This paper introduces a naturalistic observational combined process- and outcome study, implementing a dual-perspective approach through standardised pre- and post-treatment questionnaires and evaluating changes in the therapeutic alliance after each session. Further, semi-structured qualitative interviews aim to illuminate the personal experiences of patients and therapists. The primary objective of the presented study is to discern whether symptoms markedly decrease following therapy. A significant secondary goal is to trace the therapeutic alliance’s evolution from both patient and therapist viewpoints, emphasising the alliance-outcome association and gender dynamics within the pairs. This paper discusses the project’s feasibility after three years and shares key insights.

**Discussion:**

Recruitment for this study has posed substantial challenges due to psychotherapists’ concerns regarding data protection, extensive documentation, and philosophical reservations about the study design. Consequently, we recruited fewer participants than initially planned. Despite these hurdles, qualitative data collection has shown notable success. Given psychotherapists’ busy schedules and reluctance to participate, more potent external incentives or a legal obligation may be necessary to encourage participation in future studies.

## 1 Introduction

Psychotherapy is a universally recognised and effective strategy for treating mental disorders (1–3). Over time, an extensive body of research has emerged, focused on scrutinising psychotherapy’s impact on informing healthcare policies and refining psychotherapeutic practices (4). Most psychotherapy research focuses on randomised controlled trials (RCTs), aiming to evaluate interventions under ideal, controlled conditions. However, this approach tends to overlook the complexities of routine clinical practice, where outcomes may differ. Critics argue that RCTs operate in environments deviating significantly from real-world psychotherapy and bypass fundamental aspects of everyday therapeutic practices. For example, RCTs often exclude patients with co-morbidities, a common occurrence in routine practice, and patients in RCTs typically lack autonomy in choosing their therapists (5–7). Furthermore, RCTs tend to rely heavily on manual-guided therapy, which could limit spontaneous interventions, possibly compromising the therapeutic relationship (8).

In light of these limitations, there is growing attention towards studies conducted in real-life conditions, known as naturalistic outcome studies. These studies offer an alternative perspective to existing RTCs by tracking individuals undergoing therapy in their everyday environments and documenting their progress over time (9). They provide a grassroots method for building and applying scientific knowledge, helping bridge the gap between science and clinical practice, often cited as an “empirical imperialism” problem. This problem arises when there is a divide between clinical practice and research. In many cases, clinicians may not be involved in research or may feel that the findings of controlled studies do not apply well to their day-to-day work with patients. This disconnect can lead to a lack of valuable input from clinicians in the research process (10). Recent years have witnessed an uptick in uncontrolled naturalistic outcome studies in Germany (11), Switzerland (12), Norway (4), and Denmark (13), and one is currently underway in Brazil (14). Austria lacks representative data on psychotherapy outcomes under the conditions of outpatient psychotherapy in outpatient practice under routine care conditions.

This article introduces a study focused on the processes and outcomes of psychotherapies under clinically representative conditions in Austria. The study’s design considers the learnings from a previous unsuccessful attempt by a different research team. The former attempt, which involved exclusively behavioural therapists, failed due to inadequate resources, limited stakeholder involvement, insufficient incentives, and a prevailing belief among therapists that further practice studies were unnecessary (15). The POPP study (Process and Outcome in Private Practice) (16) detailed in this paper welcomes participation from all psychotherapeutic modalities acknowledged by Austrian psychotherapy legislation. Consequently, it encompasses therapeutic modalities from four major theoretical orientations: Humanistic therapy, Psychodynamic therapy, Systemic therapy, and Behavioural therapy. The POPP study is supported by a team of postdoctoral researchers funded by universities. It ensures a more realistic study duration and broad stakeholder involvement, thereby addressing the limitations encountered in the earlier trial. While we have built upon lessons learned, we needed to revise our initial expectations regarding the number of study participants. Through this publication, we aim to 1) describe the planned methodology and rationale of the research project and 2) explain the ongoing challenges we face in conducting a naturalistic outcome study in Austria despite incorporating lessons learned from a previous attempt.

## 2 Background

Evidence-based psychotherapy is fundamentally based on collections of empirically supported treatments (ESTs) (16). These provide clinicians access to treatments validated through robust research, typically through RCTs. However, these collections primarily concentrate on differential efficacy, determined by the therapeutic modality used in treatment. Despite more than half a century of research, countless RCTs, and substantial monetary investment, the effects of specific psychotherapy modalities on mental disorders have proven to be only moderate (3). The focus on the efficacy of a therapeutic modality may also skew the broader perspective, as it overlooks substantial evidence indicating that the effectiveness of treatment largely hinges on the person of the therapist (a) (17), the complex dynamics of the therapy relationship (b) (18), and on the person of the patient (c) (17, 19).

Therapist variables (a), which include technical and relational skills, responsiveness, and attentiveness to the patient’s emotional experiences, contribute significantly to the outcome (17, 20–22). Research indicates that therapists who excel at forming strong therapeutic alliances often achieve better treatment outcomes (23). The overall magnitude of therapists’ effects tends to be greater in naturalistic settings than in RCTs (24). The therapeutic alliance (b), defined by the bond between the therapist and patient, agreed therapeutic objectives, and collaborative tasks, is pivotal for patient transformation during psychotherapy (25–27). This dynamic aspect of therapy, which evolves over time, is frequently viewed as a predictor of therapeutic outcomes. Even though the therapeutic alliance’s correlation with the outcome is not too high (*r*=.28), various studies underscore its significance (28). In this context, the specific and common factors models of psychotherapy are worth mentioning. While the specific factors model assumes that ingredients specific to each therapy approach contribute most to therapy outcome (e.g., specific therapeutic interventions), the common factors model assumes that factors realised in all psychotherapies contribute most to the outcome (e.g., the therapeutic relationship or alliance). The specific factors model has also been named the medical model of psychotherapy, and the common factors model has been developed into the contextual model of psychotherapy (29). However, Cuijpers et al. (2019) (30) argue that no specific or common factor in psychotherapy has been sufficiently researched or theorised to be recognised as an evidence-based mechanism of change. According to research from a decade ago, patients and their psychotherapists hold disparate views concerning the therapeutic alliance and its aspects. Patients tend to attribute greater importance to factors such as support, joint engagement in the therapeutic process, and potential strain in the alliance. At the same time, therapists often concentrate on goals and tasks (31). However, this understanding of differing perspectives has been challenged and expanded by recent studies and meta-analyses. Current research indicates a strong correlation between the evaluations of the alliance by patients and therapists, particularly in adult populations. For instance, Flückiger et al. (2018) (28) provide a comprehensive meta-analytic synthesis demonstrating this alignment in adult psychotherapy, emphasising the interconnectedness of patient and therapist perspectives in forming a successful therapeutic alliance. Similarly, Laws et al. (2017) highlight the convergence in therapeutic alliance ratings between patients and therapists and its significant relation to treatment outcomes in chronic depression (32). On the other hand, several studies in naturalistic settings examining whether congruence is linked with different types of outcomes have yielded mixed results (33).

Several patient factors (c), too, have been demonstrated to predict treatment outcomes, including age, gender, ethnicity, partnership status, comorbidity, self-reported disability, and personality disorder traits, among others (17, 19, 34). While Luborsky et al.’s early literature review (1971) (35) suggested better outcomes when therapist and patient genders align, Flückiger et al.’s (2018) above-mentioned meta-analytic synthesis on the alliance in adult psychotherapy (28) concluded that individual patient characteristics, including gender, generally have a small impact on treatment outcomes compared to other factors, such as the therapeutic alliance. This suggests that while gender may play a role in psychotherapy outcomes, it is likely overshadowed by the quality of the therapeutic relationship and other elements of the therapeutic process. However, while gender might influence the outcome only minimally, there are nuanced ways gender can influence therapy. For example, a study analysing data from over 17,000 students and 200 therapists at a US university counselling centre from 1996 to 2008 found that male patients engaged in longer treatment periods with male therapists, with comparable results to their female counterparts (36). Also, a preference has been observed among female patients for same-gender therapists, particularly for sensitive topics (37, 38). A study involving over 300 patients demonstrated that some therapists, more than others, can work more effectively with one gender over another. Some psychotherapists achieved better outcomes working with female patients, while others had more success with male patients. However, for some therapists, no significant difference was observed in their effectiveness with patients of different genders (39). One Swiss study showed that female therapists’ contributions were perceived as more supportive, while male therapists were viewed as more analytical (40). Austrian research involving 1,357 patients linked risky developments in therapy primarily to male-therapist-female-patient pairings (41). Qualitative insights suggest gender influences therapist-patient dynamics, with young female therapists sometimes feeling uneasy with older male patients due to flirtation and confrontation proving more difficult in female-female pairs. Male-male pairs tend to experience early rivalries; however, once a good relationship is established, it becomes easier for male patients to discuss topics such as sexuality. The study also identifies topics that are more challenging to address in mixed-gender dyads: sexuality, desire, and other issues related to corporeality (42). Such dynamics impact the therapeutic process, highlighting the importance of understanding gender’s role in therapy. While existing research provides some understanding, it does not elucidate the reasons behind observed differences (36), neglects critical aspects of the therapy process, such as the evolving therapeutic relationship (40), or lacks depth in exploring phenomena related to different gender combinations. Combining quantitative and qualitative data in a mixed methods design is necessary to acquire a comprehensive understanding, ensuring both therapist and patient perspectives are represented (43).

## 3 Design and methods

### 3.1 Aims and research questions

The main objective of the POPP study is to evaluate the *outcome and process* of psychotherapies amidst the complexity and fluidity of naturalistic conditions over time at varying lengths. A distinct emphasis is placed on examining the therapeutic relationship, focusing on the collaborative and trustful bond between a therapist and a patient (the alliance), and gender dynamics. Importantly, the study also includes data from patients who end their therapy prematurely for various reasons, offering an inclusive and thorough perspective on the psychotherapeutic process. In particular, the POPP study will answer the following research questions:

1) Given an open-ended, naturalistic, and representative psychotherapy setting (outpatient psychotherapy practice), what are the rates and magnitudes of diagnostic, symptomatic, and interpersonal change (the outcomes gauged through pre- and post-therapy comparisons)?
2) How does the therapeutic alliance evolve in outpatient psychotherapy practices from the viewpoints of both patients and therapists? This includes contrasting these dual perspectives within each therapeutic orientation and potentially even within individual therapeutic modalities, provided sufficient data exists. For a discrete evaluation of a therapeutic modality, a minimum of ten patient-therapist pairs are required.
3) How does the therapeutic alliance connect to the therapy’s outcome from both the patient’s and therapist’s standpoints?
4) How are therapy outcomes influenced by therapist demographics (e.g., age, gender), professional experience, personality traits, and the congruence of therapist-patient characteristics?
5) Which interventions are perceived as beneficial or detrimental to the psychotherapy process from the perspectives of patients and therapists?
6) What impact does the gender pairing within the therapeutic dyad (female-female, male-female, male-male, female-male) have on the therapeutic alliance, therapy outcome, or premature termination of therapy?

It is crucial to clarify that the POPP study does not aim to juxtapose the efficacy of the four theoretical orientations or their numerous therapeutic modalities. Due to the inherent design of a naturalistic outcome study, there is no control group, and participants are not assigned randomly to different treatment modalities. Consequently, there might be intrinsic variations amongst individuals who opt for different therapeutic modalities. These variations impede direct comparisons (44).

### 3.2 Setting

The POPP study, designed to reflect the prevalent psychotherapy conditions in Austria, offers an authentic insight into the field. Austria, an early adopter of psychotherapy legislation, enacted a law in 1991 (BGBL 361/1990) defining psychotherapy as a scientifically-based treatment for behavioural disorders and psychological suffering on a psychosocial or psychosomatic basis (45). The study operates within Austria’s mental health system, featuring 23 accredited psychotherapeutic modalities categorised into four broader theoretical orientations: humanistic, psychodynamic, systemic, and behavioural. As of 2021 (46), the distribution of psychotherapists according to their theoretical orientation was as follows: 38% practised Humanistic therapy, 26% were Psychodynamic therapists, 24% practised Systemic therapy, and 12% were Behavioural therapists.

The Austrian model exemplifies an uncommonly diverse blend on a global scale and sets Austria apart from regions dominated by only a few mainstream modalities, such as psychoanalysis and behavioural therapy. Notably, this model provides an array of entry pathways and multiple training opportunities in psychotherapy (47, 48). The POPP study allows us a closer look into, and a deeper understanding of, the particular characteristics of Austria’s unique psychotherapy model.

### 3.3 Design

Launched in 2020, the POPP project utilises a sophisticated mixed-methods research design, synergising quantitative and qualitative methodologies. It involves a wide-ranging participant pool, encompassing patients with various and combined symptoms and undergoing treatment durations ranging from as brief as three sessions to as extended as four years. Furthermore, the study aims to incorporate these patients’ corresponding psychotherapists to align and compare their data.

The quantitative part of the study involves pre- and post-therapy online surveys taken by patients and their therapists. Both groups are also requested to assess the therapeutic alliance after each session, enabling real-time process monitoring. Therapists document their cases using a specific case report format.

The qualitative part of the study encompasses interviews with patients and their respective psychotherapists. These interviews occur randomly at several therapy stages, either at the initial, the mid-point, or the concluding phases, whereby individuals are only asked once during the process. These interviews, guided by a semi-structured interview guide, probe deeply into individual experiences.

During the analysis process, quantitative and qualitative data forms corroborate each other. The qualitative data contribute valuable insights into the psychosocial subtleties and dynamics of therapy that might potentially elude the quantitative data. The quantitative analysis will primarily scrutinise the relationship between the therapeutic alliance and therapy outcomes across different gender pairings, considering the perspectives of both patients and therapists. Furthermore, the qualitative data, in an independent capacity, will shed light on the subjective experiences of patients and therapists alike.

**Figure 1:**
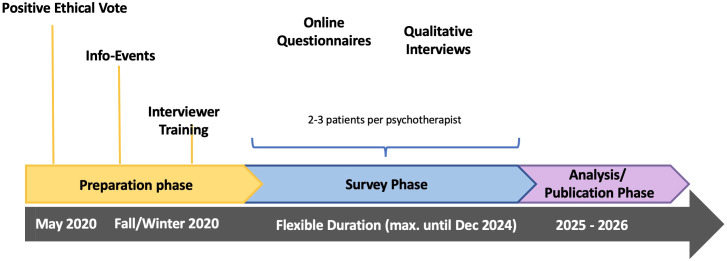
Research procedure. The survey follows the course of psychotherapy and ends individually with its conclusion. It includes various processes with different lengths, therapeutic approaches, and symptoms.

This research project represents a collaborative endeavour involving the University of Continuing Education Krems (UWK) and the Karl Landsteiner Private University for Health Sciences in Krems. Research approval for the study was obtained from the Data Protection Office of the University of Continuing Education Krems (UWK) as well as from the Ethics Committee of the University of Continuing Education Krems (UWK), Austria (EK GZ 28/2018-2021). The timeline of the study can be accessed in **Figure 1**.

### 3.4 Recruitment of participants

The success of the POPP study hinges on psychotherapists actively participating and being ready to include two to three of their patients. Psychotherapists eligible for involvement must be officially licensed and listed in the Austrian Federal Ministry for Social Affairs, Health Care, and Consumer Protection’s directory, demonstrating their qualification to provide individual outpatient psychotherapy to adult clients in their psychotherapy practice; group therapies are not incorporated in this study.

To ensure a broad and representative sample of patients, psychotherapists are instructed to consecutively invite all their patients in the nascent therapy stages between their first and third sessions to partake in the study. They should invite on a consecutive basis without bias towards the severity, type of symptoms, or personal preferences. Psychotherapists must record the reasons if an invited patient either declines participation or is not extended an invitation, thereby aiding in detecting recruitment biases.

The study aimed to register a minimum of 100 therapists and 300 patients to secure representation across all four theoretical orientations and the 23 therapeutic modalities recognised in Austria. In the spirit of collaboration and inclusive research, all professional therapeutic associations in Austria have been invited by our research team to contribute a minimum of ten psychotherapists each, to facilitate individual data evaluation of their respective therapeutic modalities.

Numerous professional psychotherapy associations, including the Austrian Federal Association for Psychotherapy (ÖBVP), assist with recruitment by disseminating information via emails, newsletters, and social media. The research team has also briefed therapists at various scientific meetings and congresses.

A central aspect of our recruitment strategy involves arranging multiple online information workshops throughout the year. These workshops aim to demystify the research design, clarify the role of the psychotherapists, and provide information about incentives. Psychotherapists who choose to participate in the study will receive a certification of five training hours per patient per year, which will count towards the obligatory 150 hours of further training over a five-year period, as mandated by Austrian law.

This study presents a unique opportunity for psychotherapists to contribute to the research community, examine and potentially improve their therapeutic practice by reflecting on their therapeutic alliance after each session, and reinforce the effectiveness of their therapeutic modalities, especially those currently lacking substantial empirical support.

Psychotherapists interested in participating in the study are encouraged to contact our research team for further information and workshop dates. Basic information about the study is also available online, hosted by the Karl Landsteiner Private University^1^. The dates and the link to the online info workshops are also provided via a Facebook site^2^.

Upon contacting the research team via the designated email address to request study materials, psychotherapists receive comprehensive informational resources in digital format or as printed materials. These resources contain thorough details about the study, handouts intended for the patients, and case report forms. Patients are then invited by their therapists to participate in the study and provided with these informational materials.

The psychotherapist’s and the patient’s materials include secure hyperlinks to the Research Electronic Data Capture (REDcap) platform (49), where they can complete quantitative surveys after granting their electronically informed consent. Patients interested in contributing to the study’s qualitative interviews can sign up through a separate online contact form.

### 3.5 Quantitative measures

#### 3.5.1 Data protection and security

Once patients register via REDCap, they are asked to create a code composed of the first letter of their last name and birth date (e.g., M1790). This code, along with the name of the treating psychotherapist, is provided by the patients as well as by the therapist in each online questionnaire. Using this method, the data from different measurement points and individuals (patient/psychotherapist) can be linked, allowing for a more streamlined data management process.

#### 3.5.2 Patient questionnaires at the beginning of psychotherapy

The research protocol includes a series of standardised questionnaires to gather information on various parameters. Demographic data such as age, gender, level of education, marital status, and occupation are collected. Additionally, data concerning the funding of the psychotherapy sessions, utilisation of psychopharmacological medications, instances of sick leave, or work incapacity due to mental disorders are collected. Another inquiry assesses patients’ awareness of the therapeutic method utilised by their psychotherapist. To assess therapy pre-post outcome the following measures are used:

Primary outcome: Positive Mental Health Scale (PMH) (50)

Secondary outcomes: Patient Health Questionnaire (PHQ-9) (51), Generalised Anxiety Disorder Scale (GAD-7) (52), and the World Health Organisation Disability Assessment Schedule (WHODAS) (53).

To explore gender aspects, the Social Roles Questionnaire (SRQ) (54) is implemented. Personality dimensions are assessed using the Big Five Inventory Short version from the Socio-Economic Panel (BFI-SOEP) (55) and the Short-Form Personality Traits Assessment (SASPD) (56). Lastly, patient expectations are examined through the Outcome Expectations Scale (OES) (57).

#### 3.5.3 Therapists’ questionnaires at the beginning of psychotherapy

We gather demographic data from therapists and use the BFI-SOEP (55) and SRQ (54) tools to evaluate their personality dimensions and gender aspects in the context of their social roles.

#### 3.5.4 Assessment of the helping alliance and change

The alliance is assessed after each psychotherapy session using the Helping Alliance Questionnaire (HAQ) (58–60). The HAQ includes two versions: one for patients and one for therapists. Each version consists of items that participants rate based on their perceptions of the therapeutic alliance. Eleven questions cover aspects such as the degree of agreement on therapy goals, the helpfulness of the therapy, and the level of mutual trust and respect. In the context of this study, patients and therapists are asked to complete the HAQ (59) after each psychotherapy session. This ongoing assessment allows for the tracking of changes and fluctuations in the therapeutic relationship over time and can provide insight into the role this relationship plays in the overall therapy outcome. The maximum likelihood estimation (ML) will be used to handle missing data in multilevel models. We chose the HAQ over other more frequently used instruments, such as the WAI (61), because it is better validated in German than other instruments, especially because the therapist version is also validated (60, 62). We also plan to use the HAQ as a measure of change during therapy, as it captures changes in well-being after each session compared to the start of therapy. Since the HAQ includes two different scales: (a) assessing satisfaction with the therapeutic relationship and (b) evaluating satisfaction with therapy outcomes, with a primary focus on treatment outcome satisfaction, the second scale (b) will also be used to track changes from the beginning to the end of therapy.

#### 3.5.5 Patients’ questionnaires at the end of psychotherapy

At this stage, we utilise a range of standardised questionnaires to assess the following outcomes for patients. These encompass the PMH (50), the PHQ-9 (51), the GAD-7 (52), and the WHODAS (53). The personality dimensions are analysed using the SASPD (56). In this phase, we also investigate the therapeutic modality implemented during the psychotherapy sessions, as assessed by the Multitheoretical List of Therapeutic Interventions instrument (MULTI 30) (63). Furthermore, any negative effects are recorded using the Negative Effects Questionnaire (NEQ) (63). Periodically, we send emails to therapists, requesting them to remind their patients about completing the questionnaires during the course of therapy. We will analyse the missing data, including the number and percentage of missing data points, the number and percentage of variables with missing data, and the number and percentage of participants with missing data. We will further clarify whether the missing data is missing completely at random, missing at random, or not missing at random and whether the missing data patterns are monotone or intermittent. Based on this assessment, we will decide whether to remove cases with missing data. Full information maximum likelihood and multiple imputation are possible options for estimating missing data if the assumptions are met (e.g., missing completely at random) and are generally preferred over casewise deletion (64, 65).

#### 3.5.6 Therapists’ questionnaires at the end of psychotherapy

To ensure comprehensive data collection, psychotherapists involved in the study complete a Case Report Form for each consecutively admitted patient until they secure two to three patients for the study. This form is submitted to the research team upon completion of the study. It includes details such as the therapist’s name, patient code, distribution and receipt of study materials, patient participation status, and reasons for non-participation, if applicable. It also records diagnoses, session dates, supervision details, treatment conclusions, reasons for non-standard treatment termination, and study discontinuation. This Case Report Form captures pertinent data during psychotherapy and streamlines the completion of the Psychotherapist Questionnaire at the end of treatment by pre-populating it with relevant data.

As the instruments used vary between psychotherapists and patients, **Table 1** provides an overview delineating which tools are employed with each group.

**Table 1:** delineates the specific instruments employed for patients and therapists.

	Start of treatment	After each session	End of treatment
**Patient**	Demographic information, PMH, PHQ-9, GAD-7, WHODAS, SRQ, BFI-SOEP, SASPD, OES	HAQ (P)	PMH, PHQ-9, GAD-7, WHODAS, SASPD, MULTI, NEQ
**Therapist**	Demographic information, BFI-SOEP, SRQ	HAQ (T)	Case report form

#### 3.5.7 Quantitative analysis

In our study, we plan to utilise t-tests for dependent samples to assess changes in psychological distress through pre- and post-intervention comparisons. Additionally, we intend to employ multilevel models to analyse the dynamics of therapeutic relationships over multiple measurement points. Multilevel models will also be applied to explore potential predictors of therapy outcomes; both patient and therapist variables will be considered.

### 3.6 Qualitative measures

#### 3.6.1 Qualitative interviews

Unlike the general study, where therapists are enrolled first, the recruitment for additional qualitative interviews initiates with patients already participating. Patients who consent to the quantitative survey can further opt to participate in a qualitative interview. They can register to be interviewed face-to-face by filling out a secure contact form on the UWK’s homepage, including their phone number, email, and county. Each time after completing their post-session questionnaire to assess the helping alliance (HAQ) (59), patients receive comprehensive information about the possibility of registering for a face-to-face interview in REDCap. Furthermore, psychotherapists involved in the study are encouraged to inform their patients about the opportunity to partake in qualitative interviews.

This study’s interview methodology delves into patients’ and therapists’ subjective impressions of the therapeutic process and the therapeutic relationship. Using a semi-structured qualitative interview approach (52), therapists and patients share their experiences by answering reflective and open-ended questions face-to-face with interviewers recruited from psychotherapy students at the UWK. The interview guide, designed for both patients and psychotherapists by BS, YS, and AJ, starts with motivations at the beginning of therapy (66). It explores the evolution of therapy goals from start to present and prompts reflection on pivotal moments and impactful dialogues within the therapeutic process. Moreover, it aims to elicit insights into what participants perceive as the most advantageous facets of therapy and probes their interpretation of the transformations and achievements they attribute to the therapy process. Notably, the guide emphasises the therapist’s role and delves into the implications of gender dynamics within the therapeutic relationship. Finally, the interview facilitates the expression of any hardships or obstacles that participants may have encountered throughout their therapy, encouraging an open dialogue about the challenges inherent in the therapeutic relationship and process.

With the therapy process’s different stages in mind, the study team has developed specific interview guides for each phase: First from the initial session to the fifth, then mid-therapy starting with the sixth session until the end of therapy and in instances of therapy discontinuation. While the patient and therapist interview guides maintain a similar thematic focus, each is uniquely tailored to capture the distinct perspective of the therapy process from the viewpoint of the patient or therapist. In this way, we aim to collect interviews from all stages of the therapy (beginning, middle, and end).

Before the interviews, patients are provided with an information form and a privacy statement, and written consent for participation is secured. This also includes the patient’s consent for their therapist to be interviewed. Upon receiving approval, the same interviewer approaches the therapist to conduct a complementary interview on the therapeutic process. These follow-up interviews ideally occur within two weeks of the patient interview to capture the same phase of the process. Therapists, similar to patients, receive a privacy statement before their interview, and their written consent for participation is obtained.

#### 3.6.2 Qualitative analysis

Our methodology involves dividing the sample into distinct sub-samples. These groups are defined based on different stages of the therapeutic process, such as early, middle, and late stages. Additionally, we consider gender as another critical factor, dividing the sample by gender or gender pairings, which allows us to examine dynamics within same-gender dyads and mixed-gender dyads. The primary objective of our qualitative analysis is not to generalise our findings to broader populations. Instead, we focus on developing detailed maps of variation within the data. These maps aim to capture and reflect the diverse practices and experiences related to the phenomena under study (67). For instance, this approach enables us to explore variations in how patients seek therapists in private practice (66) or their views and experiences with therapists of the same or opposite gender. We also closely examine therapists’ perspectives, including their views and experiences with interventions or what patients and therapists find helpful at different stages. To extract these nuanced insights, our analysis relies on category-based methods in qualitative research. This approach is instrumental in identifying recurring patterns and themes across the data, encompassing patients, therapists, dyads or subgroups in terms of stages or gender.

#### 3.6.3 Data collection process and data management

A crucial collaborative aspect of this project is the inclusion of psychotherapy students at the UWK in the research process with the aim to train scientist-practitioners. This will provide the students with an early-career model based on conducting scientifically rigorous and clinically relevant studies. It will help them identify clinically relevant questions later in their work (22). The UWK, offering postgraduate training in psychosocial interventions, involves their Master’s students from multiple psychotherapy programs in data collection and analysis. These students, working towards their certification as academically trained psychotherapists under the Austrian Psychotherapy Act (BGBL 361/1990), can utilise project data for their Master’s thesis in return for their involvement. These students are recruited during research seminars and via notifications to those enrolled in the University’s psychotherapy programs. Interested students are briefed about the study in an informational session, after which they sign a confidentiality agreement.

When patients participating in the quantitative survey also confirm their interest to partake in a qualitative interview, a Master’s student contacts a few patients (typically three to four) to schedule interviews at a location chosen by the patients. After the interview, they seek the patient’s consent to interview their psychotherapist.

As they conduct and transcribe the interviews independently, students receive supervision from staff members available for questions. We also offer focus groups and troubleshooting support. These interactions are essential for identifying and addressing methodological challenges and refining interview guidelines.

The gathered data is stored on a secure university drive and shared collectively with the students. Each case folder must contain consistent documents: an interviewer’s memo, an audio file, a transcript, and a signed consent form. We also keep a keyword list in an Excel file documenting the interview contents, ensuring uniformity in data format and digital document presentation. Depending on their Master’s thesis project requirements, students can create their subsets of data after submitting their collected data to the pool.

#### 3.6.4 Focus groups

Qualitative data collection was conducted by two separate waves of students, who later engaged in focus group discussions. This process was established to oversee the research procedure and maintain the quality of the findings. These focus groups, led by two staff members, provide a platform for interviewers to discuss their insights regarding the process’s feasibility and their overall interview experiences. As of now, two waves of data collection and their corresponding focus group sessions have been completed.

## 4 Recruitment process

### 4.1 Qualitative and quantitative data

**Figure 2:**
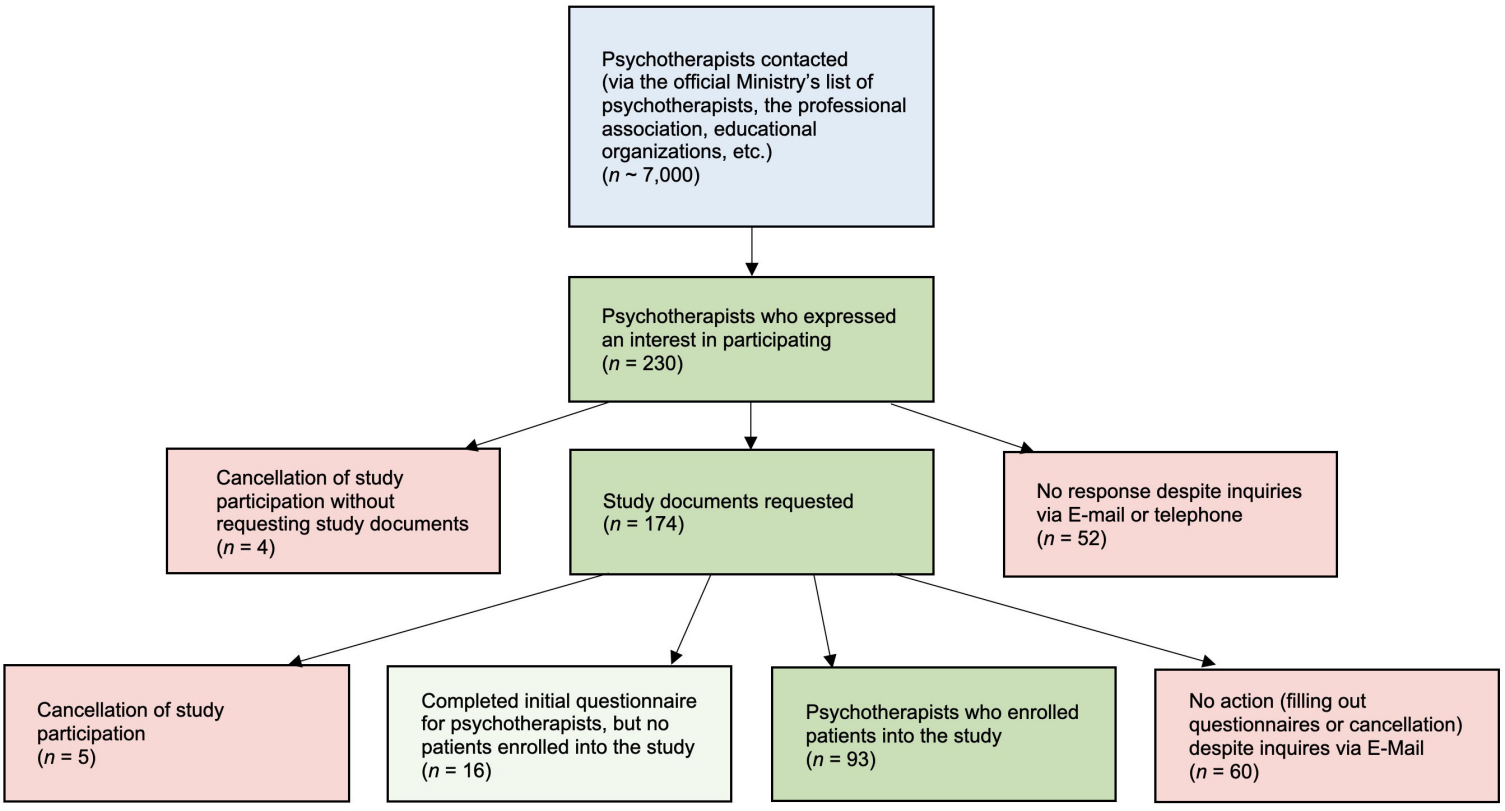
Overview of the recruiting process for psychotherapists.

Despite our extensive efforts and incentives to motivate psychotherapists to participate in the study, the recruitment process has emerged as the most significant challenge, as can be seen in **Figure 2**. By now, we have acquired data from 93 therapists and 173 patients, and we are far from realising the goal of securing a minimum of 10 psychotherapists for each therapeutic modality. The study’s conclusion, initially scheduled for 2023, has been deferred to December 2024 due to the slow pace of data collection. This means we included new psychotherapists in the study until mid-2023 and collect data until the end of 2024. The therapy, of course, goes on even after the study is finished. Most psychotherapists were admitted in the first year, and the average psychotherapy time in private practice of 2-4 years was considered in our planning.

Contrary to the difficulties experienced in the broader recruitment for the study, soliciting qualitative interviews has been noteworthy successful. Over the course of two waves carried out in the summers of 2021 and 2022, we have successfully completed 43 patient interviews and 41 psychotherapist interviews. During the initial wave, 6 students conducted interviews with 21 patients and 17 therapists. This was followed by a second wave, where an additional 8 students interviewed 22 patients and another 17 therapists. Notably, a subset of therapists was interviewed twice, with each occasion focusing on a distinct patient.

The comprehensive dataset thus compiled is particularly impressive, given that these interactions occurred amidst the constraints posed by the COVID-19 pandemic.

### 4.2 Difficulties with incomplete data and data management procedures

While we have collected over 3,452 process surveys from therapists until today (January 2024), patient submissions amount to 1,990. Missing process survey data from patients underscores the importance of having a simple and user-friendly process for data collection. Furthermore, we assume that a stronger commitment to the study on the part of the therapists would also have led them to regularly remind their patients to enter the data for the process survey. This happened too little.

One frequent difficulty encountered is patients’ inconsistent usage of codes when they login to complete the questionnaires compared to when the interviewers contact them via phone. This inconsistency hampers the data merging process. We partly ascribe these issues to the pathology patients grapple with. This pathology also introduced challenges for the interviewers, as voiced by one of them during a focus group:

*“Occasionally, the pathology influenced the dynamics of the interviews in a bizarre way. For instance, it was unclear whether one patient was speaking in metaphors or experiencing psychosis. That was quite draining.” (researcher 3, focus group 1)*

However, there have been instances where psychotherapists failed to use consistent codes as well, indicating that mental pathology cannot be the sole reason for such confusion.

### 4.3 Working with student researchers

Working with student researchers presented great advantages and only minor challenges due to their diverse experiences with scientific research and qualitative methodologies. While many have social or health sciences backgrounds, others have never worked in a scientific field, impacting the interview process and data quality. However, due to their psychotherapy training, these student researchers are adept at conversational techniques. They have learned to form a strong rapport with their counterparts and reflect on the information they receive. All of them were already working with patients under supervision, which positively influences the openness of interviewees and the depth of conversation. However, during focus groups, it became apparent that students often struggled to differentiate between their roles as therapeutic interviewers and researchers. They found it challenging to disengage from a therapeutic conversation and follow the interview guide.

During a focus group, one researcher expressed,

*“I constantly had to remember, ‘ah, here’s my questionnaire, and I’m here as a researcher.’ Sometimes it would have been emotionally appropriate to ask questions or summarise, but then there was the INTERVIEW GUIDE. I felt as if I was juggling multiple roles at once.” (researcher 1, focus group 1)*

A recurrent challenge faced by our student researchers pertains to the expansive geographical scope of participant recruitment, encompassing all regions of Austria. As a result, students often needed to travel significant distances to interview patients and psychotherapists. Despite our endeavours to align the students’ places of residence with the locations of our study participants, such coordination was not always feasible.

## 5 Discussion

### 5.1 Recruitment of participating psychotherapists

Even though Austrian law requires a scientific approach to psychotherapy, a 2018 study found Austrian therapists to be more resistant to process and outcome monitoring than their counterparts in Belgium, the UK, and Australia. These therapists expressed apprehension about complex procedures, the perceived minimal treatment benefits, and potentially detrimental effects on the therapist-patient relationship (68). As we already mentioned in the introduction, similar problems were encountered in previous psychotherapy research efforts (15).

Despite our comprehensive and strategic efforts, which encompass refining our research approach, dedicating significant human resources, and actively involving stakeholders (7) in the context of the POPP study, we persistently encounter challenges that impede our recruitment process. Throughout the series of 22 informational workshops from October 2020 to June 2023, we have identified several concerns expressed by potential participants that could discourage their involvement:

Concerns about the study have been notably focused on data protection and potential disruption of the therapeutic process. Therapists worry about guaranteeing their patients’ confidentiality and the possible negative effects of ongoing data collection, particularly post-session questionnaires, on their therapeutic relationships. They also expressed concerns about interpreting individual data and gauging their therapeutic efficacy on a personal level.

There were also concerns about the strain filling out questionnaires and process monitoring puts on patients.

The complexity of the study information, as necessitated by data protection regulations, emerged as a further barrier to participation. Therapists expressed doubts about their patients’ capacity to understand such intricate details. Furthermore, the study’s complex design was occasionally misconstrued as a randomised controlled trial (RCT), evoking apprehensions about reducing psychotherapeutic processes to symptom-oriented constructs. Particularly, therapists from humanistic modalities emphasised their focus on less tangible aspects like personality development and enhancement of self-perception, contending that these elements could not be accurately captured through standardised questionnaires. In this vein, previous research on the attitudes of Austrian psychotherapists on process and outcome monitoring has found that cognitive-behavioural therapists generally held favourable views; therapists employing humanistic-existential modalities, on the other hand, expressed negative attitudes (68). This attitude is anchored in broader, epistemological objections to psychology’s characterisation as a quantitative science and its aspiration to produce “objective” explanations for psychological phenomena (69). Despite these findings, however, most participating psychotherapists belong to humanistic psychotherapy modalities.

Despite our consistent efforts during the workshops to clarify that our study did not aim to compare the effectiveness of various therapeutic modalities, we probably could not alleviate these concerns sufficiently. Given Austria’s endorsement of 23 psychotherapy modalities and the upcoming revision of the Psychotherapists Act, there is a prevalent sense of unease about potential regulatory changes. Particularly, smaller professional associations expressed fears that their modalities may not be deemed sufficient, thus jeopardising their future inclusion. This, coupled with a perceived sense of inferiority against larger, mainstream approaches such as behavioural therapy, likely dissuades therapists from participating in research more broadly.

We think the efforts to explain our study design during workshops were largely unsuccessful due to a widespread unfamiliarity with the concept of scientific research. As pointed out by Castonguay et al. (2014), many clinicians had not had the opportunity to work with a supervisor who was both a practising psychotherapist and a researcher. This gap in early career training means they lacked exposure to a role model who could identify the most appropriate research methods to answer the most relevant research questions (10).

The intensive documentation needed for participation was another deterrent, in line with findings from Laireiter and Kopetzky (2014) (15).

Some therapists expressed challenges in persuading new patients to join the study. They felt it was inappropriate to ask patients to contribute additional effort for the sake of psychotherapy, especially since the patients were already paying for the therapists’ services. However, therapists who firmly believed in the study’s value reported facing no issues enlisting patients. This indicates that the therapists’ personal hesitations might serve as a barrier to motivating patient involvement in the study.

Qualitative data collection proceeded more smoothly than quantitative data, indicating less therapist resistance. This inference comes from the noticeable increase in qualitative interview registration following email communication urging therapists to encourage their patients to participate in qualitative interviews.

Finally, unlike many therapists, patients probably did not share the same apprehensions concerning data protection, the complexities of the study’s framework, or the affiliated duties. Additionally, they value the distinctive introspective possibility offered by these personal interviews. Almost unanimously, every patient approached by our student interviewers for a deeper explanation regarding qualitative interview participation (after registration for the qualitative survey via RedCap) agreed to participate, with only a few exceptions.

### 5.2 Data management procedures

The issue with patients and therapists using different codes when logging in and during interviews indicates a need for clearer communication and an easier procedure for creating a patient code.

### 5.3 Working with student researchers

The challenges interviewers face, as reflected in the focus group sessions, highlight the need for support systems for the interview team. Handling challenging situations, such as deciphering whether a patient is speaking in metaphors or experiencing psychosis, can be draining. Since our student interviewers are being trained as future psychotherapists, they did handle such situations well. Moreover, since we provided regular sessions for reflection and support via email and telephone, they could address pressing issues immediately.

From the outset, the project prioritised data quality, aiming to build a robust database to facilitate a wide array of future research inquiries. Several quality assurance strategies have been implemented to ensure this high-quality data, including:

- Providing methodological training and support materials for student interviewers.
- Initiating early data documentation using templates to capture the extensive research context surrounding the interview situation.
- Ensuring opportunities for interviewer reflexivity.

Standardisation of the management of qualitative data proved to be advantageous. Given the complexity of a longitudinal mixed-methods dataset, understanding the data structure, familiarising oneself with documentation routines, and tracking documents for deposition and their scheduled dates can be challenging. We thus recommend having at least one person oversee data management, processing, and archiving (70).

### 5.4 Limitations

Certain limitations should be acknowledged. Despite comprehensive efforts to enhance the feasibility of the POPP study, certain limitations impacting the recruitment process persist. The resistance among Austrian psychotherapists, influenced by concerns about data protection, potential disruption of the therapeutic process, and the strain on patients, poses a significant feasibility challenge. The complexity of study information, particularly related to data protection regulations, further hampers participant understanding and engagement. Efforts to clarify the study design during workshops faced difficulties due to widespread unfamiliarity with scientific research concepts among therapists. The study’s sample was comprised only of therapists interested in the study, thus limiting its generalizability. Although therapists were advised to inform all consecutive patients about the study, adherence to this protocol couldn’t be verified. Hence, the possibility of therapists inviting only a specific subset of patients, such as those with less severe illnesses, cannot be discounted, which could potentially limit patient-side generalizability. Further limitations include missing data, especially among patients. While the baseline assessments experienced the least amount of missing data, there was a noticeable increase in missing data in the session-based questionnaires and those conducted post-therapy. Although we periodically send emails to therapists, requesting them to remind their patients about completing the questionnaires during the course of therapy, a significant amount of data will be missing, which needs to be handled appropriately based on the final percentage of missing data as well as the distribution of missing data. Possible inaccuracies due to the use of erroneous codes by patients or therapists represent an additional limitation, which could affect the interpretation of study results.

Another limitation regards the collection of qualitative interviews. Our objective is to gather qualitative interviews from all stages of therapy (beginning, middle, and end). However, realising this goal presents certain challenges. A key limitation is the unpredictability of each patient’s therapy duration. This uncertainty is evident when conducting interviews, as we cannot foresee the total length of the therapy process for each patient. Additionally, the process of conducting interviews is time-intensive, and given our limited personnel resources, it becomes impractical to accurately manage the distribution of interviews across different therapy phases. Therefore, these factors combined hinder our ability to evenly distribute the collection of interviews throughout the various stages of therapy. Consequently, we observe a predominance of interviews originating from the middle phases of therapy. This imbalance restricts our ability to compare experiential patterns characteristic of early therapy stages, late stages and processes with premature termination, limiting the scope of our analysis.

A notable limitation arises in the collection of consistent outcome measurements during the practice study, primarily stemming from the challenges posed by varying start times and the decentralised locations of patients. The nature of real-world practice settings introduces a level of unpredictability, making it difficult to ensure uniformity in the timing of outcome measurements across different treatment trajectories.

## 6 Conclusion

Despite employing various strategies and incentives to engage psychotherapists in the POPP study, participant recruitment has proven to be a significant challenge. The slower-than-anticipated data collection rate has led us to extend the study’s deadline to December 2024. The reluctance of Austrian psychotherapists to participate can be attributed to practical concerns, such as data security and extensive documentation, as well as philosophical disagreements with the study’s design and quantitative research methods. The above problems suggest that many therapists are unfamiliar with up-to-date methodological and statistical advances because they are not trained in research. Revision of the Austrian law for psychotherapy could change that.

Given the demanding schedules of psychotherapists, dedicating sufficient time to meet the requirements of a naturalistic outcome study is daunting. Although it was posited that Austria’s therapeutic diversity could be maintained only if each of its 23 therapeutic modalities substantiates its effectiveness, most modalities have not motivated at least ten therapists to participate. This suggests that more robust external motivators are needed to increase participation in future nationwide studies evaluating psychotherapy’s effectiveness. Implementing a legal mandate for participation in scientific studies could significantly augment Austria’s psychotherapy research data. Another approach could involve exposing young psychotherapists in training to research during their studies to foster enthusiasm and engagement in research. Enriching their scientific training in conjunction with patient work may provide additional benefits.

Ultimately, examining outpatient psychotherapy within the context of a therapist’s practice confronts many challenges, primarily attributable to the restricted control over participants compared to clinical trials. Given these conditions, therapists’ roles transform into critical conduits for disseminating information about the study to patients, a role typically played by the hosting institution in a conventional clinical trial setting. This dynamic might provide a credible rationale for the paucity of such essential studies in the field. Despite these complexities, we maintain optimism that the results will contribute to a deeper understanding of the processes and outcomes of psychotherapies under the condition of routine outpatient care in Austria.

## Ethics statement

The studies involving humans were approved by Ethics Committee of the University of Continuing Education Krems (UWK), Austria (EK GZ 28/2018-2021). The studies were conducted in accordance with the local legislation and institutional requirements. The participants provided their written informed consent to participate in this study.

## Author contributions

YS: Conceptualization, Data curation, Writing – original draft, Investigation, Writing – review & editing. AJ: Conceptualization, Data curation, Writing – original draft, Writing – review & editing. EH: Data curation, Investigation, Methodology, Project administration, Writing – review & editing. KH: Visualization, Writing – review & editing. CP: Writing – review & editing. TP: Methodology, Writing – review & editing. BS: Methodology, Visualization, Writing – original draft, Writing – review & editing.

## Footnotes

1
https://opencampus.kl.ac.at/de/system/files/Pr%C3%A4sentation_POPP%20f%C3%BCr%20Homepage.pdf

2
https://www.facebook.com/POPPStudie
